# Inhibition of effector B cells by ibrutinib in systemic sclerosis

**DOI:** 10.1186/s13075-020-02153-8

**Published:** 2020-03-30

**Authors:** Jakob Einhaus, Ann-Christin Pecher, Elisa Asteriti, Hannes Schmid, Kathy-Ann Secker, Silke Duerr-Stoerzer, Hildegard Keppeler, Reinhild Klein, Corina Schneidawind, Joerg Henes, Dominik Schneidawind

**Affiliations:** 1 Department of Hematology, Oncology, Clinical Immunology and Rheumatology, University Hospital Tuebingen, Otfried-Mueller-Str. 10, 72076 Tuebingen, Germany; 2 Centre for Interdisciplinary Clinical Immunology, Rheumatology and Autoinflammatory Diseases, University Hospital Tuebingen, Tuebingen, Germany

**Keywords:** Systemic sclerosis, B cells, Ibrutinib, Autoimmune disease, Treatment

## Abstract

**Objective:**

Systemic sclerosis (SSc) is a connective tissue disease with a significant morbidity and reduced survival of patients. Effective treatment and clinical control of the disease remain challenging. In particular, the development of pulmonary and cardiac fibrosis and pulmonary hypertension are severe complications responsible for excessive mortality. Currently available treatment strategies only alleviate symptoms and slow disease progression. Here, we investigated the therapeutic potential of ibrutinib, a Bruton’s tyrosine kinase (BTK) inhibitor used in B cell malignancies, to alter B cell pathology in SSc in an in vitro model of autoimmunity.

**Methods:**

PBMCs and sorted B cells of 24 patients with SSc were used for functional testing after stimulation with hypomethylated DNA fragments (CpG) to induce an innate immune response. The effects of ibrutinib on cytokine production, autoantibody release, and activation of the transcription factor NFκB were evaluated.

**Results:**

Ibrutinib was able to reduce the production of the profibrotic hallmark cytokines IL-6 and TNF-α mainly from the effector B cell population in patients with SSc. Importantly, small doses of ibrutinib (0.1 μM) preserved the production of immunoregulatory IL-10 while effectively inhibiting hyperactivated, profibrotic effector B cells. In a flow cytometry analysis of phosphorylated NFκB, an important transcription factor in the induction of innate immune responses in B cells, significantly less activation was observed with ibrutinib treatment.

**Conclusion:**

Our data could pave the avenue for a clinical application of ibrutinib for patients with SSc as a novel treatment option for the underlying pathogenetic immune imbalance contributing to disease onset and progression.

## Introduction

Systemic sclerosis (SSc) is a connective tissue disease that affects the skin, blood vessels, and internal organs. Within a complex and incompletely understood pathogenesis, the defining pathophysiological features include immune dysregulation with the production of autoantibodies, vasculopathy, and chronic activation of fibroblasts. In particular, the involvement of internal organs critically reduces survival of patients [1]. A stratification of patients in risk-associated groups in the recently developed SCleroderma mOrtality p Eustar (SCOpE) score allows for precise prediction of 3-year mortality, estimating the survival of high-risk patients (SCOpE ≥ 15) at 53% [2]. More than half of the patients die from SSc itself, mostly due to cardiopulmonary complications [3]. Thus, currently available treatment options summarized in the updated EULAR recommendations remain insufficient for controlling disease progression in a clinically satisfactory way [4].

The pathogenesis of SSc remains poorly understood. Accumulating evidence suggests that B cells are involved in SSc beyond the mere production of autoantibodies. Alterations to the B cell compartment maintain the hyperreactivity and chronic activation of large portions of the immune system. The sensitive equilibrium between effector B cells and regulatory B cells (Bregs) is disrupted, and immunoregulatory Bregs prove to be numerically and functionally impaired [5]. Furthermore, evidence for an important pathogenetic role for effector B cell-derived profibrotic IL-6 and TNF-α, as well as for protective effects of anti-inflammatory IL-10, has been published recently [6].

Consequently, different approaches have been tested to address the hyperactivation of B cells in SSc. In a phase III study, the IL-6-receptor-α inhibitor tocilizumab failed to reduce skin thickening, but a trend toward improvement of the modified Rodnan Skin Score and pulmonary function was observed [7]. Complete B cell depletion with rituximab showed better efficacy with regard to the reduction of skin fibrosis and respiratory restriction in a case-control study [8]. The clinical improvement in both trials might have been limited by the lack of specificity, as depletion of all B cell subsets eliminates the protective effects conveyed by Bregs in the context of autoimmunity.

The specific inhibition of autoreactive, profibrotic, and chronically activated B cell subsets represents a more promising approach to achieve effective treatment of SSc. The Bruton’s tyrosine kinase (BTK) inhibitor ibrutinib could inhibit hyperactivated B cell subpopulations to counteract underlying pathogenetic mechanisms. First FDA-approved for mantle cell lymphoma in 2013 and chronic lymphocytic leukemia in 2014, a potential application for ibrutinib has been suggested for any autoimmune disease in which B cells play an important role [9, 10]. Here, we report on the potential of this small molecule inhibitor to alter B cell pathology in primary patient samples in order to pave the avenue for a clinical application of ibrutinib in patients with SSc.

## Materials and methods

### Patients and healthy volunteers

Peripheral blood samples were collected from patients with SSc enrolled at the Centre for Interdisciplinary Clinical Immunology, Rheumatology and Autoinflammatory Diseases at the University Hospital Tuebingen, Germany, from 2017 to 2019. Written consent was obtained from all patients. Human buffy coats from healthy volunteers were obtained from the Center of Clinical Transfusion Medicine Tuebingen. The institutional review board of the Eberhard-Karls-University Tuebingen (IRB approval number 114/2016BO) approved this study to be in accordance with the ethical standards and the Helsinki Declaration of 1975, as revised in 2013.

### Magnetic cell separation

B cells were purified from cryopreserved peripheral blood mononuclear cells (PBMCs) of patients with SSc using CD19-Microbeads (Miltenyi Biotec, Bergisch Gladbach, Germany), a QuadroMACS™ Separator (Miltenyi Biotec) and LS Columns (Miltenyi Biotec) according to the manufacturer’s instructions. After purification, cells were cultured in a medium consisting of RPMI 1640 GlutaMAX™ Medium (ThermoFisher Scientific, Waltham, USA), 10% human serum (C-C-Pro, Oberdorla, Germany), 100 IU/ml penicillin-streptomycin (Lonza, Basel, Switzerland), 5.5 μM 2-mercaptoethanol (Roth, Karlsruhe, Germany), 0.1 mM non-essential amino acids (Gibco, New York, USA), 10 mM HEPES (Gibco), and 1 mM sodium pyruvate (Gibco).

### Cytokine analysis

For cytokine profiling, B cells were preincubated with ibrutinib (Selleckchem, Houston, USA) or 0.1% DMSO (control) for 1 h at a concentration of 2 × 10^6^ cells/ml. B cells were then stimulated with the Toll-like receptor 9 agonist CpG (1 μM, ODN2006, Invivogen, San Diego, USA) and cultivated on a 96-well-plate for 24 h at a concentration of 1 × 10^6^/ml. Supernatants were collected and stored at − 20 °C prior to analysis in a multiplex assay (LEGENDplex™ Mix-and-Match-Panel 5-plex, BioLegend, San Diego, USA) according to the manufacturer’s instructions. For data analysis, the LEGENDplex™ Software v8.0 from BioLegend was used.

### Anti-Scl-70-ELISA

Supernatants of B cell cultures were collected after 72 h and stored at − 20 °C until further analysis. Nunc polystyrene plates (ThermoFisher Scientific) were coated with recombinant human DNA topoisomerase (Scl-70, Diarect AG, Freiburg, Germany) overnight. Anti-Scl-70 antibodies were detected in undiluted culture supernatant using peroxidase-conjugated anti-human IgG H + L (goat, Jackson ImmunoResearch Laboratories Inc., West Grove, USA).

### Intranuclear staining of PBMCs

Patient PBMCs were thawed and preincubated with ibrutinib (Selleckchem) or 0.1% DMSO (control) for 1 h at a concentration of 2 × 10^6^ cells/ml. The TLR9-agonist CpG (0.1 μM, ODN2006, Invivogen) was added to PBMCs cultivated on a 24-well-plate (1 × 10^6^/well). After 24 h, cells were stained for CD3 (OKT3, BV605, BioLegend) and CD19 (HIB19, BV421, BioLegend). Fixable Viability Dye eFluor™ 780 (eBiosience, Thermo Fisher Scientific) was used for the identification of live cells. The cells were fixed with IC Fixation Buffer (eBiosience, Thermo Fisher Scientific) and permeabilized with 90% ice-cold methanol. To detect phosphorylation levels of NFκB (Ser536), a phospho (p) NFκB antibody (93H1, Cell Signaling, Boston, USA) was stained with a secondary anti-rabbit antibody (PE-Cy7, Cell Signaling). Samples were measured using an LSR II Fortessa flow cytometer (BD Biosciences, Franklin Lakes, USA).

### Intracellular cytokine staining

PBMC cultures were performed as described. A cell stimulation cocktail (eBioscience, Thermo Fisher Scientific) was added to the culture for the last 4 h of culturing. After 24 h, cells were stained with Fixable Viability Dye eFluor™ 780 and the following antibodies purchased from BioLegend or BD BioScience: CD3 (HIT3a, PerCP-Cy5.5), CD20 (2H7, BV510), CD24 (ML5, BV650), CD27 (O323, BV421), CD38 (HIT2, PE/Dazzle), and IgD (IA6-2, FITC). Fixation and permeabilization were performed using an IC Fix and Perm Buffer Kit (eBioscience, Thermo Fisher Scientific). Anti-human IL-6 (MQ2-13A5, PE-Cy7) was used to detect intracellular cytokine levels in B cells.

### Statistical analysis

Flow cytometry data were analyzed in FlowJo V10 (Tree Star Inc., Ashland, USA). Prism 7.01 (GraphPad Software, La Jolla, USA) was used for further statistical analysis and graphical representation. Experiments were performed in technical duplicates and repeated independently at least three times. Significance (*p < 0.05, **p < 0.01, ***p < 0.001) was calculated using a paired Student’s *t* test for single comparisons and ANOVA testing for repeated measures for multiple comparisons.

## Results

### Patient characteristics

In total, samples of 24 patients were used for in vitro testing (Table 1). The median age of the patient cohort was 54 years (range 30–81), and the median disease duration after diagnosis was 8 years (range 1.2–24). Most patients were tested positive for antinuclear antibodies (ANA), and half of the study cohorts were positive for anti-Scl-70 antibodies. The median modified Rodnan skin score was 8. Importantly, 75% of patients received no immunosuppressive treatment at the time of blood draw. Six patients indicated prior intensive immunosuppressive regimens in their patient history.

**Table 1:** Patient characteristics

***SSc (n = 24)***	
Demographics	
**Age (years)**	
Median	54
Range	30–81
**Sex**	
Female	15 (63%)
Male	9 (38%)
Disease characteristics	
**SSc subtype**	
Limited cutaneous SSc	14 (58%)
Diffuse cutaneous SSc	10 (42%)
**Disease duration (years)**	
Median	8
Range	1.2–24
**Modified Rodnan skin score**	
Median	8
Range	0–44
**Autoantibodies**	
Antinuclear antibodies (ANA)	20 (83%)
Anti-Scl-70 antibodies	12 (50%)
Anticentromer antibodies (ACA)	6 (25%)
**Pretreatment**	
Cyclophosphamide	4 (17%)
Mycophenolate mofetil	2 (8%)
Stem cell transplant (> 10 years before blood draw)	2 (8%)
**Immunosuppressive therapy at time of blood draw**	
None	18 (75%)
Prednisolone	2 (8%)
Mycophenolate mofetil	2 (8%)
Methotrexate	3 (13%)

### Reduction of proinflammatory cytokines and anti-Scl-70 in B cells through high-dose ibrutinib

Elevated cytokine levels in SSc mirror the ongoing process of chronic inflammation that contributes to fibrosis and organ destruction. We reasoned that ibrutinib would exert meaningful effects on the cytokine profiles of stimulated B cells in SSc. A number of observations support a relevant immuno-activating role for TLR9-activating double-strand self-DNA in SSc [11]. Therefore, B cells were stimulated with the TLR9-agonist CpG robustly inducing the profibrotic cytokines IL-6 and TNF-α.

**Fig. 1:**
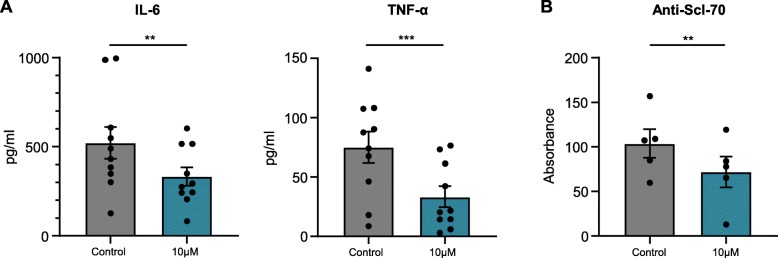
Effects of high-dose ibrutinib on the release of proinflammatory cytokines and anti-Scl-70. B cells were treated with ibrutinib (10 μM) and stimulated with CpG (1 μM); DMSO was used as control. **a** Supernatants of B cell cultures were analyzed for cytokine levels in a multiplex assay after 24 h of culture (*n* = 10). **b** Supernatants of B cell cultures were analyzed for anti-Scl-70 levels via ELISA after 72 h of culture (*n* = 5). Bars represent the mean. Error bars indicate SEM. *p < 0.05, **p < 0.01, ***p < 0.001

Cytokine levels were determined in the culture supernatant after 24 h (Fig. 1a). Here, high-dose ibrutinib treatment significantly reduced the production of IL-6 and TNF-α by B cells from 522.7 pg/ml (SEM ± 88.9 pg/ml) to 333.5 pg/ml (SEM ± 51.48 pg/ml, *p* = 0.003) and from 75.0 pg/ml (SEM ± 13.1 pg/ml) to 33.5 pg/ml (SEM ± 8.8 pg/ml, *p* = 0.0004), respectively. A similar reduction of cytokine production by B cells with ibrutinib treatment was observed in healthy volunteers (Supplemental Figure 1). Moreover, the effects of ibrutinib on autoantibody production by B cells of anti-Scl-70-positive patients were investigated after 72 h of culture (Fig. 1b). Anti-Scl-70 antibodies, or anti-topoisomerase I-antibodies, are characteristic for SSc and were produced by stimulated B cells in vitro. Ibrutinib reduced the release of anti-Scl-70 significantly from 103.7 (SEM ± 16.0) to 72.0 (SEM ± 17.3, *p* = 0.002).

### Dose-dependent modulation of the B cell cytokine profile through ibrutinib

**Fig. 2:**
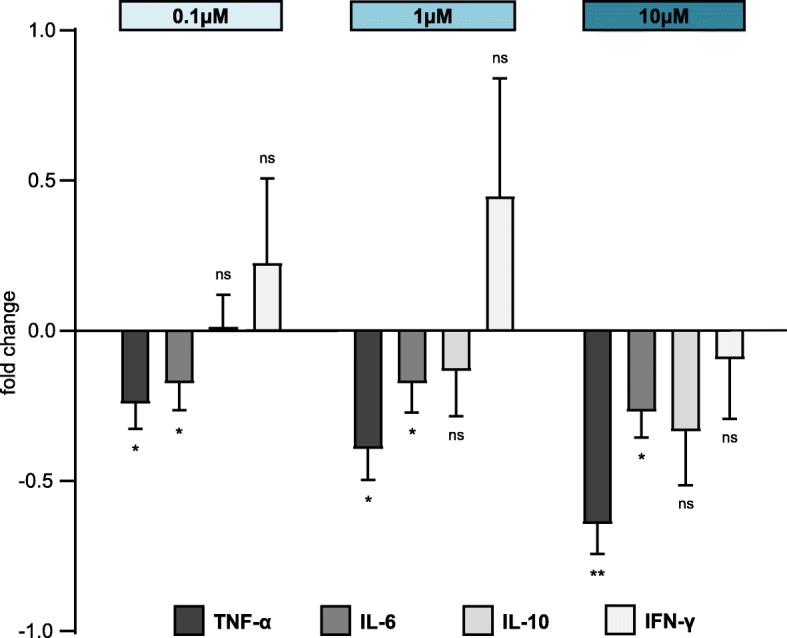
Relative changes of cytokine production under ibrutinib treatment. B cells of patients with SSc (*n* = 5) were treated with ibrutinib (0.1 μM, 1 μM, 10 μM). The relative change in cytokine production compared to control is depicted. Low concentrations of ibrutinib (0.1 μM, 1 μM) reduce TNF-α and IL-6 significantly but do not alter the level of IL-10. High concentrations of ibrutinib (10 μM) reduce cytokine production nonspecifically. Bars indicate the mean. Error bars show SEM. *p < 0.05, **p < 0.01

To further determine the clinical applicability of ibrutinib in the context of SSc, alterations to the B cell cytokine profile of patients with SSc were assessed over a spectrum of concentrations whenever allowed by the respective cell count of the samples. The relative changes of cytokine production were analyzed, comparing ibrutinib-treated and control samples (mean concentrations: IL-6 = 575.9 pg/ml, TNF-α = 95.7 pg/ml, IFN-γ = 4.9 pg/ml, IL-10 = 5.7 pg/ml) within each individual patient (Fig. 2). Lower concentrations of ibrutinib exerted effects on B cells that differed strongly from the modulation of cytokine production through high-dose ibrutinib. For high-dose ibrutinib (10 μM), a nonspecific reduction in cytokine production for all detected cytokines (IL-6 − 27.3%, TNF-α − 64.8%, IFN-γ − 9.8%, IL-10 − 33.8%) was observed. In contrast, low concentrations of ibrutinib affected the production of pro- versus anti-fibrotic cytokines in a cytokine-specific manner: the proinflammatory cytokines TNF-α and IL-6 were noticeably reduced at 0.1 μM (TNF-α − 24.6%, IL-6 − 17.9%) and 1 μM (TNF-α − 39.8%, IL-6 − 17.9%), while the immunoregulatory IL-10 remained unchanged at 0.1 μM and was only mildly decreased at 1 μM (− 13.7%). Anti-fibrotic IFN-γ even increased in ibrutinib-treated samples compared to control (0.1 μM + 23.2%, 1 μM + 45.4%). Importantly, B cell viability was not decreased with ibrutinib treatment in experiments with samples of healthy volunteers, nor was apoptosis induced in B cells upon ibrutinib treatment.

### Ibrutinib-mediated inhibition of the transcription factor pNFκB

**Fig. 3:**
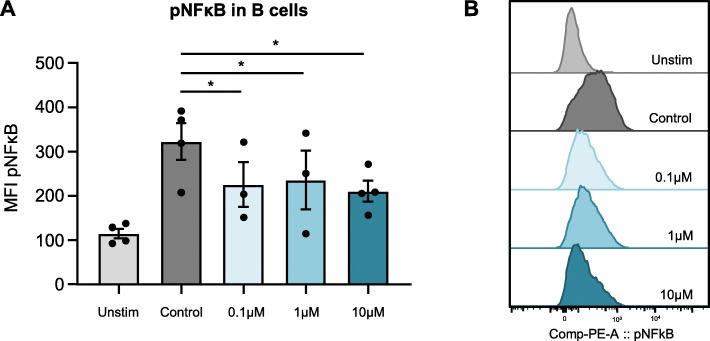
Ibrutinib-mediated inhibition of the transcription factor NFκB. PBMCs of patients with SSc (*n* = 4) were treated with ibrutinib and stimulated with CpG (0.1 μM) for 24 h; DMSO (0.1%) was used as control. Phosphorylated NFκB (Ser536) was stained after methanol permeabilization. **a** Levels of pNFκB (MFI) are reduced significantly by ibrutinib. Bars depict the mean. Error bars represent SEM. *p < 0.05. **b** Representative histograms from one individual SSc patient

NFκB is a transcription factor with a major role in downstream TLR and B cell receptor (BCR) signaling, conveying survival signals and inducing a proinflammatory response in B cells [12, 13]. Flow cytometry analysis of the phosphorylation of NFκB was performed to measure the transcriptional activation downstream of TLR9 after 24 h. CpG stimulation increased the abundance of pNFκB (Ser536) by 2.82-fold compared to unstimulated B cells (Fig. 3a, b). With ibrutinib treatment, a significant reduction in the level of phosphorylation was observed (0.1 μM, *p* = 0.047; 1 μM, *p* = 0.029; 10 μM, *p* = 0.018).

### Reduction of IL-6^+^ naïve (CD27^−^) B cells in SSc PBMCs treated with ibrutinib

**Fig. 4:**
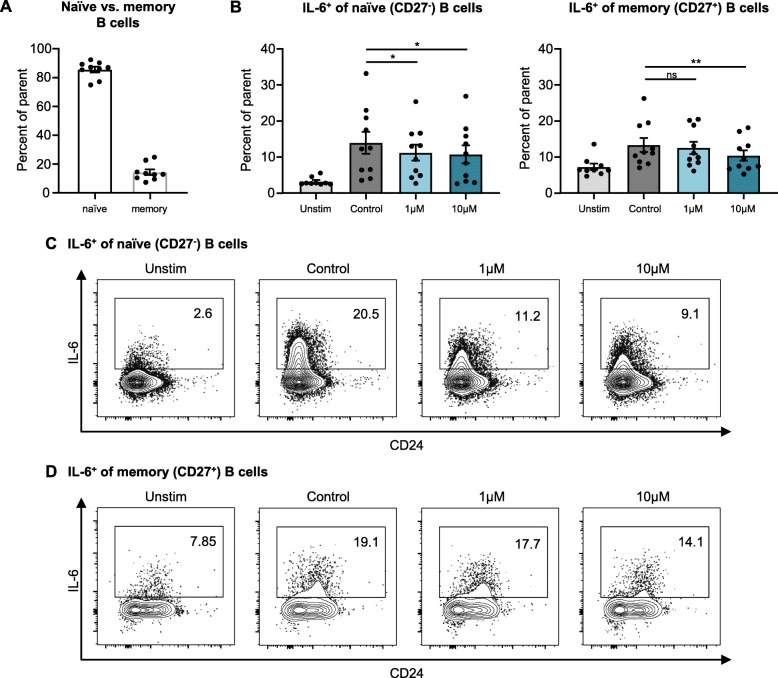
Subset analysis of IL-6 production via intracellular cytokine staining. PBMCs of patients with SSc (*n* = 10) were incubated with CpG (0.1 μM) for 24 h under ibrutinib treatment; DMSO (0.1%) was used as control. **a** Percentage of naïve and memory B cells of all B cells in unstimulated controls. **b** Ibrutinib treatment reduces the amount of IL-6^+^ naïve (CD27^−^) B cells significantly at low dosages, while no significant change was observed in the subset of memory B cells (CD27^+^). Bars represent mean. Error bars indicate SEM. *p < 0.05, **p < 0.01. **c** Representative dot plots of naïve (CD27^−^) B cells. **d** Representative dot plots of memory (CD27^+^) B cells

To evaluate the effect of ibrutinib on different subpopulations of B cells, patient PBMCs were cultured over 24 h and stained for intracellular IL-6. In our patient cohort, the ratio of naïve (CD27^−^) to memory (CD27^+^) B cells was consistently shifted toward naïve B cells (Fig. 4a): in unstimulated samples, naïve B cells accounted for 85.6% (SD ± 5.6%) of all B cells while memory B cells represented only 14.4% (SD ± 5.8%). Neither ibrutinib treatment nor stimulation with the TLR9-agonist CpG altered the ratio of the B cell subpopulations significantly (data not shown). At baseline in unstimulated samples, a higher proportion of memory B cells (7.3%, SD ± 2.6%) was IL-6^+^ compared to naïve B cells (3.3%, SD ± 1.1%) (Fig. 4b). Ibrutinib (10 μM) showed a significant reduction in the proportion of IL-6^+^ cells within both naïve and memory B cells. In contrast, 1 μM of ibrutinib significantly decreased the IL-6^+^ proportion of naïve B cells (Fig. 4b, c) but showed no significant effect on memory B cells (Fig. 4b, d).

## Discussion

Ibrutinib (IC_50_ = 0.5 nM for BTK inhibition) is a first-in-class, irreversible inhibitor of the BTK that can effectively inhibit BCR signaling by selective active-side binding [14]. The BCR pathway plays an important role in B cell development and survival, as it regulates proliferation, differentiation, and apoptosis [15]. Targeting BCR signaling holds the prospect of significantly altering diseases with pathological B cell activation and proliferation. Consequently, ibrutinib was first tested in the context of B cell malignancies and proved effective in patients with relapsed or refractory non-Hodgkin’s lymphoma, chronic lymphocytic leukemia, or Waldenström macroglobulinemia [16]. Importantly, ibrutinib as a single agent showed proficient tolerability and a low side-effect profile [9, 10, 17]. On the other hand, while a beneficial impact of ibrutinib in autoimmune diseases like rheumatoid arthritis or SSc has been proposed from the very beginning, the transition to a clinical application in this field is incomplete [18].

In SSc, B cells are assumed to be important players in disease onset and progression [19, 20]. B cells of patients with SSc show elevated expression levels of the regulatory surface molecule CD19, which reduces the threshold of BCR signaling, thereby importantly influencing B cell activation and survival [21]. This overexpression of CD19 has been linked to the production of SSc-specific autoantibodies, as well as increased levels of profibrotic cytokines, and contributes to a chronically hyperactivated B cell population [22, 23]. We hypothesize that inhibition of the BTK could be the key to restoring B cell physiology and might, therefore, provide a substantial improvement to SSc treatment.

While the BCR is composed of a unique antigen-specific immunoglobulin binding a certain epitope, TLRs recognize a variety of molecular patterns associated with pathogens or cell damage. Signaling downstream of TLR9, a member of the TLR family recognizing unmethylated single-strand DNA, is known to be augmented in SSc, supporting collagen deposition from fibroblasts and synergizing with BCR signaling for B cell activation and immunoglobulin class-switching [11, 24]. It is suggested that circulating fragments of self-DNA acting as endogenous TLR9-ligands could have a role in SSc development and progression [25, 26]. In our in vitro model, the TLR9-agonist CpG (ODN2006) increased survival of cultured B cells and induced the production of various cytokines and anti-Scl-70-antibodies. While no indications of increased B cell apoptosis were observed, ibrutinib treatment showed convincing potential to counteract the production of key inflammatory cytokines, specifically IL-6 and TNF-α. Both cytokines play pivotal roles in the perpetuation of fibrotic signaling leading to skin thickening and organ fibrotic transformation [27, 28]. As a single agent, ibrutinib combines the effects on IL-6 and TNF-α and could alter the production of more cytokines involved in the pathogenesis of SSc as well. A central transcription factor downstream of TLR9 signaling inducing the production of these inflammatory agents is NFκB. Our flow cytometry analysis of phosphorylated NFκB shows increased activation of TLR9 signaling even 24 h after stimulation. Ibrutinib reduced the abundance of pNFκB significantly, supporting previous findings that describe BTK as a key signaling molecule of the TLR9 pathway [29]. The reduction of anti-Scl-70 under ibrutinib treatment is further encouraging and might be true for other autoantibodies. Even though autoantibodies are more of a diagnostic marker than a prognostic factor in SSc, a contribution to disease development via immune activation cannot be ruled out [30]. In vitro findings suggest that anti-Scl-70-antibodies could be of direct pathogenetic relevance by binding to the cell surface of fibroblasts [31].

In our model, ibrutinib was able to reduce the release of proinflammatory IL-6 and TNF-α at doses even below the effective concentrations achieved in vivo (Fig. 2). Importantly, physiologically applicable doses of ibrutinib showed a biased inhibition of fibrogenic cytokines while maintaining IL-10 and IFN-γ levels. Serum IL-10 levels are generally not decreased in patients with SSc, but a significant reduction in IL-10-producing B cells has been described [5, 32, 33]. The preservation of the anti-fibrotic effects of IL-10 under ibrutinib treatment is considered important in the context of aggravated skin fibrosis in a B^IL10−/−^ mouse model by Matsushita et al. [6] On the other hand, the role of interferon type II (IFN-γ) is controversially discussed in SSc. Some describe a reduction of serum IFN-γ and a pathogenic imbalance of Th1 and Th2 cytokines [33], while others found elevated IFN-γ production without any correlation to clinical outcomes [34]. Functionally, IFN-γ as a Th1-cytokine is categorized to have anti-fibrotic effects and might be reactionarily increased in patients with SSc in an attempt to control fibrotic transformation [35]. Thus, an increase in IFN-γ under ibrutinib treatment could contribute to restore the physiological Th1-Th2-balance.

In a flow cytometric analysis of intracellular cytokines, ibrutinib inhibited the production of IL-6 preferentially in the naïve (CD27^−^) B cell subpopulation. A reduction of IL-6^+^ memory (CD27^+^) B cells was restricted only to high-dose ibrutinib treatment (10 μM). In SSc, the composition of the B cell population is pathologically altered. Memory B cells are reduced and show a high susceptibility for apoptosis-inducing signals, while naïve B cells are numerically increased [20, 36]. Treatment with ibrutinib for 24 h did not change the relative ratio of B cell subsets, but the biased inhibition of IL-6 production represents an indicator for a differential influence of ibrutinib on B cell subpopulations that could translate to meaningful differences between subsets in survival and activation in vivo.

## Conclusion

In summary, our in vitro data could pave the avenue for a clinical application of ibrutinib as a novel treatment of the underlying pathogenetic immune imbalance in SSc. Overall, we provide convincing evidence that ibrutinib has the potential to improve B cell pathology contributing to disease onset and progression, encouraging a clinical translation to the benefit of patients with SSc.

## Supplementary information

**Supplemental Figure 1.** Cytokine production by B cells of healthy volunteers with ibrutinib treatment (*n* = 4). B cells of healthy volunteers were stimulated with CpG for 24 h and treated with DMSO (Control) or ibrutinib (10 μM). With a Legendplex assay, cytokine levels of IL-6, TNF-⍺, IFN-γ and IL-10 were determined in the culture supernatant. Bars represent mean. Error bars indicate SEM. * *p* < 0.05, ** *p* < 0.01, *** *p* < 0.001.

## Data Availability

Data are available from the corresponding author on a reasonable request.

## Abbreviations

Anti-Scl-70: Anti-topoisomerase I antibody
BCR: B cell receptor
Bregs: Regulatory B cells
BTK: Bruton’s tyrosine kinase
CD: Cluster of differentiation
DGRh: German Society for Rheumatology
DMSO: Dimethylsulfoxid
ELISA: Enzyme-linked immunosorbent assay
FDA: Food and Drug Administration
SSc: Systemic sclerosis
IC50: Half-maximal inhibitory concentration
IFN-γ: Interferon gamma
IL: Interleukin
MFI: Mean fluorescence intensity
ns: Nonsignificant
PBMCs: Peripheral blood mononuclear cells
pNFκB: Phosphorylated nuclear factor kappa-light-chain-enhancer of activated B cells
SD: Standard deviation
SEM: Standard error of the mean
Th: T helper
TLR: Toll-like receptor
TNF: Tumor necrosis factor
