# Supplementary material for: Inhibition of effector B cells by ibrutinib in systemic sclerosis
Source: Arthritis Res Ther. 2020 Mar 30;22:66. doi: 10.1186/s13075-020-02153-8 (PMC7106617; doi:10.1186/s13075-020-02153-8)
Supplement: Supplementary file 1 — Supplemental Figure 1. Cytokine production by B cells of healthy volunteers with ibrutinib treatment (n = 4). B cells of healthy volunteers were stimulated with CpG for 24 h and treated with DMSO (Control) or ibrutinib (10 μM). With a Legendplex assay, cytokine levels of IL-6, TNF-⍺, IFN-γ and IL-10 were determined in the culture supernatant. Bars represent mean. Error bars indicate SEM. * p < 0.05, ** p < 0.01, *** p < 0.001. [file 13075_2020_2153_MOESM1_ESM.pdf]

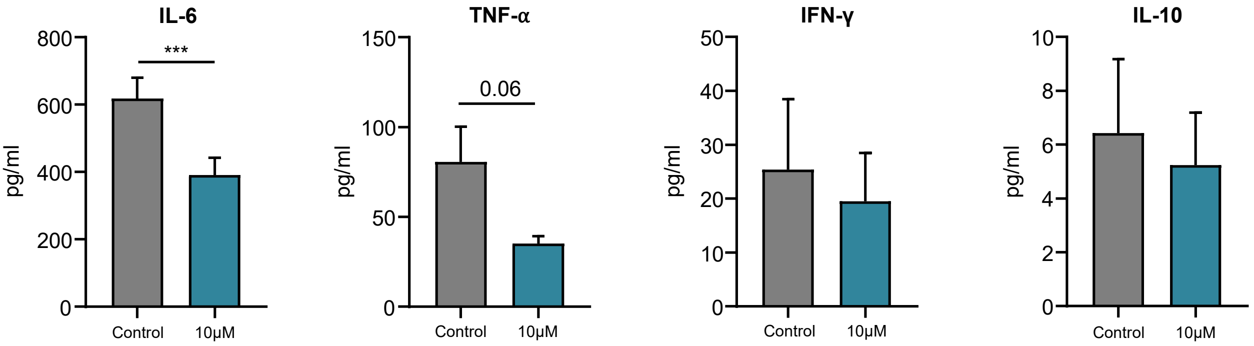

Supplemental figure 1: Cytokine production by B cells of healthy volunteers with ibrutinib treatment (n=4)
B cells of healthy volunteers were stimulated with CpG for 24h and treated with DMSO (Control) or ibrutinib (10μM). With a Legendplex assay, cytokine levels of IL-6, TNF-α, IFN-γ and IL-10 were determined in the culture supernatant. Bars represent mean. Error bars indicate SEM. * $p < 0.05$, ** $p < 0.01$, *** $p < 0.001$.
